# Noninvasive Differentiation of Indolent from Aggressive Renal Neoplasms with MR Fingerprinting Combined with Diffusion/Perfusion MRI

**DOI:** 10.1148/radiol.251404

**Published:** 2026-03

**Authors:** Sree Harsha Tirumani, Christina J. MacAskill, Yilun Sun, Dheeman Futela, Holly Harper, Adam Calaway, Bernd Kuehn, Ke Cheng Liu, Lee Ponsky, Mark Griswold, Chris A. Flask, Yong Chen

**Affiliations:** 1 Department of Radiology, Case Western Reserve University, 11100 Euclid Ave, Bolwell Bldg, Room B120, Cleveland, OH 44106; 2 Department of Radiology, University Hospitals Cleveland Medical Center, Cleveland, Ohio; 3 Department of Biomedical Engineering, Case Western Reserve University, Cleveland, Ohio; 4 Population and Quantitative Health Sciences, Case Western Reserve University, Cleveland, Ohio; 5 Department of Pathology, Case Western Reserve University, Cleveland, Ohio; 6 Department of Pathology, University Hospitals Cleveland Medical Center, Cleveland, Ohio; 7 Department of Urology, Case Western Reserve University, Cleveland, Ohio; 8 Department of Urology, University Hospitals Cleveland Medical Center, Cleveland, Ohio; 9 MR Application Predevelopment, Siemens Healthineers, Erlangen, Germany; 10 Siemens Medical Solutions USA, Malvern, Pa; 11 Department of Pediatrics, Case Western Reserve University, Cleveland, Ohio; 12 Department of Pediatrics, University Hospitals Cleveland Medical Center, Cleveland, Ohio

**Keywords:** GU, MR, OI

## Abstract

**Background:**

Accurate characterization of tumor grade and aggressiveness for renal neoplasms with noninvasive imaging methods is critical for patient management and outcome.

**Purpose:**

To evaluate kidney MR fingerprinting (MRF) alone and in combination with diffusion-weighted imaging and arterial spin labeling (ASL) MRI for the diagnosis and characterization of renal neoplasms by assessing its performance in differentiating indolent from aggressive tumors.

**Materials and Methods:**

Adults with suspected renal cell carcinoma (RCC) were recruited between November 2020 and October 2024 for this prospective proof-of-concept study. Histopathologic grading served as the reference standard, and renal masses were classified into indolent (low-grade or benign) and aggressive (high-grade or unclassified RCCs) types according to their natural biologic behavior. Noncontrast coronal T1 and T2 maps were acquired using breath-hold kidney MRF (two to five sections per participant) at 3.0 T. Respiratory-triggered diffusion-weighted imaging and ASL MRI were further applied to measure the apparent diffusion coefficient (ADC) and renal blood flow (RBF), respectively. Univariable and multivariable analyses were performed to identify parameters that differentiated renal neoplasms and correlated with histopathologic findings.

**Results:**

Renal neoplasms from 24 participants (mean age, 61 years ± 11 [SD]; 16 female) were characterized as indolent (*n* = 14) and aggressive (*n* = 10) types. A between-group difference in T2 was observed (indolent, 86 msec ± 22; aggressive, 61 msec ± 14; *P* = .005), whereas no evidence of between-group differences in T1, ADC, or RBF were observed (*P* > .05). The area under the receiver operating characteristic curve (AUC) values of T1, T2, ADC, and RBF alone for between-group differentiation were 0.39, 0.83, 0.38, and 0.62, respectively. Multivariable analysis revealed that MRF-derived T1 and T2 provided complementary information in tumor type differentiation, achieving an AUC of 0.89 in this initial exploratory analysis.

**Conclusion:**

Rapid multiparametric quantitative imaging with kidney MRF improved the ability to assess the grade and aggressiveness of renal neoplasms in 15 seconds per imaging section and without injectable MRI contrast material.

## Summary

Kidney T1 and T2 relaxation times extracted from MR fingerprinting provided complementary information for noninvasive differentiation of indolent versus aggressive renal neoplasms.

Renal cell carcinoma (RCC) is the most common malignant renal neoplasm; therefore, its management imposes a substantial economic burden on health care systems (1,2). RCC is often incidentally detected, manifesting as an incompletely characterized renal mass (3). Many patients with incidental renal masses undergo either direct surgery or biopsy without further imaging evaluation, as accurate histologic diagnosis using modern imaging techniques is not always possible. However, upfront surgery or biopsy is not ideal, as nearly 25% of incidental renal masses are indolent (benign or low-grade tumors), requiring only conservative management with active monitoring. Accurate differentiation of indolent from aggressive renal neoplasms can avoid unnecessary surgery and associated complications and help reduce psychosocial stress (4).

MRI is ideally suited for renal neoplasm assessment owing to its various soft tissue contrasts, high spatial resolution (approximately 1-mm in-plane resolution), and three-dimensional imaging capability. However, conventional MRI techniques are typically limited to qualitative interpretation based on a set of contrast-weighted images instead of actual quantitative tissue properties, which often leads to uncertain, inaccurate, and subjective image interpretation (5). Significant efforts have thus been devoted to developing novel quantitative imaging biomarkers for kidney cancer. Multiple quantitative features, including the apparent diffusion coefficient (ADC) from diffusion-weighted imaging (6–8), renal blood flow (RBF) from arterial spin labeling (ASL) MRI (9), and multivariable modeling with these parameters (10,11), have been evaluated with variable success. However, these approaches often lack method standardization, and their performance needs to be further improved for clinical applications.

One of the key limitations of these prior studies was the overall poor reproducibility of conventional quantitative MRI methods owing to multiple factors, including B_0_ and B_1_ field heterogeneities and susceptibility to motion artifacts. Invasive biopsy with sample histopathologic analysis remains the reference standard but is limited by complication risk and classification errors due to intratumoral heterogeneity. Therefore, it is desirable to have a noninvasive, objective, and reproducible imaging technique to increase the diagnostic confidence in characterizing renal neoplasms and predicting tumor grade and aggressiveness, with the goals of reducing the need for biopsy or surgery in eligible patients and selecting patients for active surveillance.

MR fingerprinting (MRF) is a multiparametric MRI method in which a rapid, highly undersampled acquisition with variable parameters is used to simultaneously generate maps of multiple MRI parameters (such as T1 and T2 relaxation times) in a single rapid MRI scan (12). Recently, MRF has been extended for multiparametric kidney imaging, and the results demonstrate that accurate T1 and T2 quantification can be achieved in a 15-second breath hold per imaging section for kidneys (13). Normative T1 and T2 values for both the renal medulla (T1 = 1827 msec ± 94 and T2 = 69 msec ± 3) and renal cortex (T1 = 1362 msec ± 75 and T2 = 64 msec ± 5) were obtained from 10 healthy individuals (13). Compared with conventional approaches, MRF has demonstrated superior performance in tissue quantification, with both improved accuracy and precision as well as overall site-to-site and scanner-to-scanner reproducibility (14–16). The ability to acquire multiple tissue properties simultaneously further enables multiparametric tissue analysis with improved accuracy for specifically identifying various pathophysiologic abnormalities (17–19). Quantitative MRI techniques, including MRF, may improve radiologists’ capability to accurately characterize renal neoplasms.

The aim of this initial proof-of-concept study was to evaluate the kidney MRF technique alone and in combination with diffusion MRI and ASL MRI for the diagnosis and characterization of renal neoplasms by assessing its performance in differentiating indolent from aggressive tumors.

## Materials and Methods

### Study Participants and Histopathologic Evaluation

In this institutional review board–approved Health Insurance Portability and Accountability Act–compliant prospective study, adults (>18 years) with suspected solid RCC tumors larger than 1 cm and no prior biopsy were enrolled at University Hospitals Cleveland Medical Center between November 2020 and October 2024. Written consent was obtained from all enrolled participants before any MRI scans were acquired. Surgical resection of the mass was performed within 2 months of the MRI examination. The surgical specimen was fixed in formalin and sectioned in the coronal plane to be consistent with MRI. Tumors were classified according to World Health Organization categories and graded on the basis of the International Society of Urological Pathology grading system, primarily according to the greatest degree of nucleolar prominence, and this histopathologic grading served as the reference standard.

Renal neoplasms were separated into two groups on the basis of RCC natural biologic behavior: the indolent group versus the aggressive group. The indolent group contained low-grade (grade 1 or 2) RCCs of any type, chromophobe RCCs, and oncocytoma, whereas the aggressive group contained high-grade (grade 3 or 4) and unclassified RCCs. Unclassified RCCs are often histologically heterogeneous and tend to be high-grade; therefore, they were included in the aggressive group in this study (20). Oncocytoma is often challenging to differentiate from hybrid oncocytic tumors and chromophobe RCCs and was therefore included in the indolent group (21).

### MRI Scan Acquisition

All MRI examinations were performed on a 3.0-T MRI scanner (MAGNETOM Vida, software version VA20A; Siemens Healthineers). The imaging protocol consisted of T1- and T2-weighted scans, kidney MRF, diffusion-weighted imaging, and ASL MRI. All these scans were acquired without the administration of a contrast agent, and the detailed acquisition parameters are listed in Table 1. The diffusion measurement was performed using the readout segmentation of long variable echo-trains (ie, RESOLVE) method with two different *b* values (0 and 400 sec/mm^2^; 12 diffusion directions). The acquisition was respiratory triggered, resulting in variable acquisition times across participants. Renal perfusion was measured with a research free-breathing pseudocontinuous ASL method with a three-dimensional turbo gradient-spin-echo readout acquisition (22). The labeling duration was 1500 msec with a flip angle of 28°. The inversion time was 3000 msec with background saturation applied across the whole kidneys.

Multiple two-dimensional kidney MRF scans (two to five sections per participant) were acquired by means of a custom-developed sequence (13). Multiple inversion recovery modules (inversion time: 20, 100, or 250 msec) and T2-preparation modules (effective echo time, 50 or 90 msec) were integrated into the MRF acquisition for effective T1 and T2 sensitivity encoding. A total of approximately 1700 MRF time frames were acquired in one 15-second breath hold for each two-dimensional MRF scan in the coronal view. Each MRF time frame was highly undersampled, with only one spiral interleaf acquired (acceleration factor, 48). Golden-angle spiral rotation was applied, and low flip angles ranging from 5° to 12° were used to minimize sensitivity to B_1_ field inhomogeneities.

### Image Reconstruction and Processing

Details of the MRF reconstruction and image postprocessing are introduced in Appendix S1. For each participant, a total of four quantitative tissue property maps (T1, T2, ADC, and RBF) were collected for data analysis. Two-dimensional region of interest (ROI) analysis was performed by an abdominal radiologist (S.H.T., with 9 years of postfellowship experience in abdominal imaging), who was blinded to the histopathologic grading, to obtain mean values for each tissue property in the solid areas of each participant’s tumor. The T1- and T2-weighted images were first reviewed to identify areas of hemorrhage and necrosis, and then the RBF maps were reviewed to identify areas of highest perfusion. ROIs were placed on the RBF maps in the region of highest perfusion, avoiding hemorrhage and necrosis to obtain the maximum perfusion value. This region was then cognitively registered on the ADC and MRF T1 maps. For the MRF measurement, the T1 and T2 relaxation time maps are inherently coregistered. ROI analysis was performed on the T1 map and automatically propagated to the T2 map to extract relaxation time values for each lesion. Interreader agreement assessment was also performed and is described in Appendix S1.

### Statistical Analysis

Descriptive statistics were used to summarize participant characteristics and imaging parameters. Data for participant age, interval between MRI and surgery, and tumor size are presented as means ± SDs or medians with IQRs. The Wilcoxon signed rank test was used to compare imaging parameters between participant groups. Logistic regression analysis was performed to evaluate the predictive performance of the four quantitative tissue properties in renal neoplasm differentiation. Univariable and multivariable analyses were performed to identify parameters that differentiated renal neoplasms. To mitigate potential overfitting given the limited sample size, bootstrap validation with optimism correction and 1000 iterations was implemented. This approach provides bias-corrected performance estimates and is appropriate for exploratory studies with limited sample sizes (23). For each bootstrap sample, the model was trained, and apparent performance metrics were calculated, whereas each test performance metric was evaluated on the corresponding out-of-bootstrap data. Optimism was calculated as the difference between the apparent and test performances for each bootstrap iteration. The mean optimism across all iterations was subtracted from the apparent performance of the model fitted on the entire dataset to compute optimism-corrected metrics, including the area under the receiver operating characteristic curve (AUC), sensitivity, and specificity. In each iteration, the optimal cutoff for sensitivity and specificity was determined at the point maximizing the Youden J index (24). The 95% CIs for these metrics were derived from the bootstrap distribution. Statistical comparison of AUCs was performed using one-sided, bootstrap-based hypothesis tests to assess whether the testing model achieved improved discriminative performance over the benchmark model, and *P* < .05 was considered indicative of statistically significant difference. All statistical analyses were conducted in R, version 4.5.1 (R Core Team, 2024), by an author (Y.S.).

## Results

### Participant and RCC Characteristics

**Figure 1:**
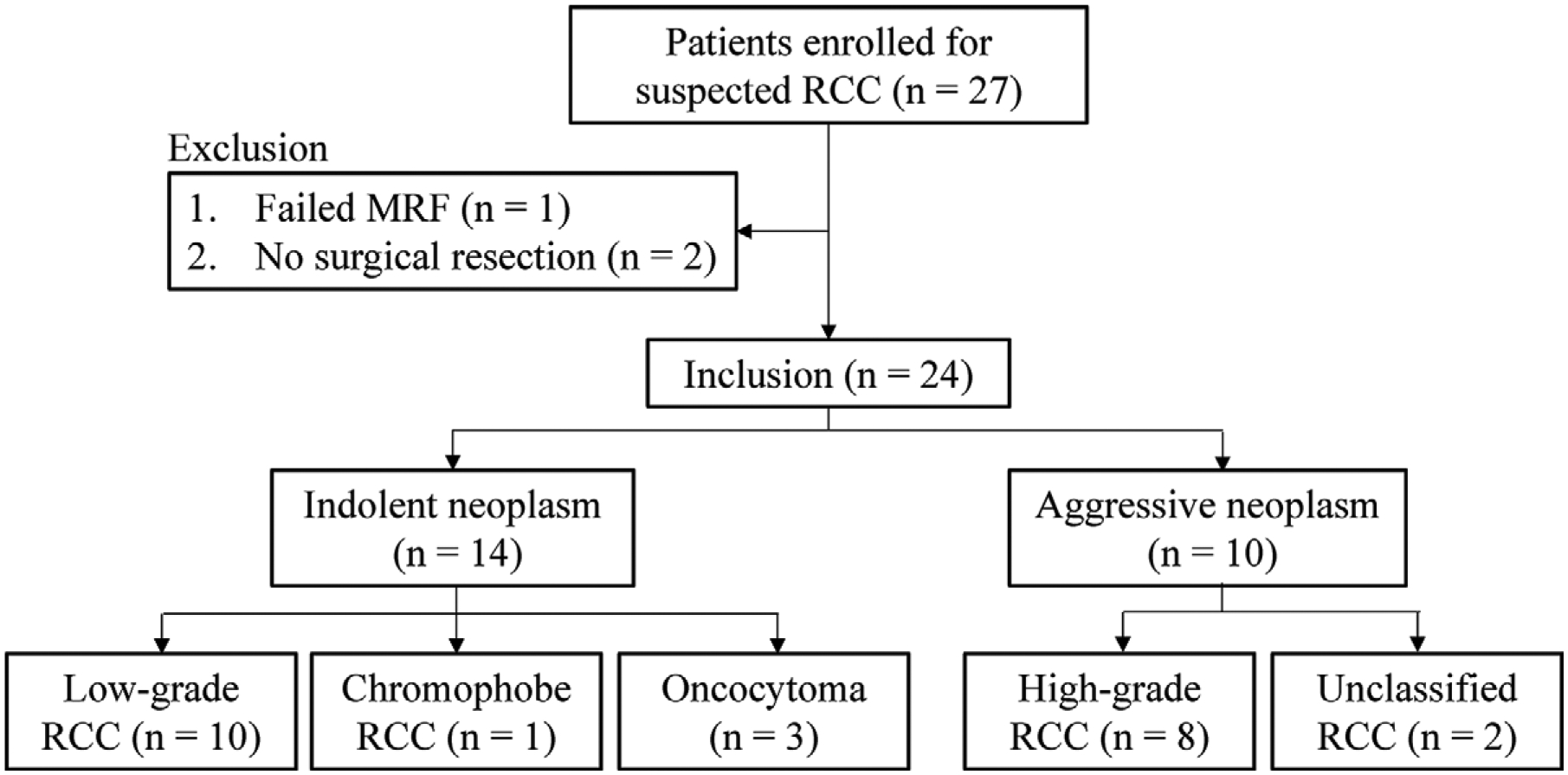
Flow diagram of participant enrollment and renal mass characteristics. MRF = MR fingerprinting, RCC = renal cell carcinoma.

A total of 27 adults with suspected RCCs larger than 1 cm were enrolled in this study. Three individuals were excluded from the data analysis because they did not undergo surgical resection (*n* = 2) or MRF failed (*n* = 1; owing to wrapped image artifacts) (Fig 1). The clinical characteristics of the 24 participants (mean age, 61 years ± 11 [SD]; 16 female and eight male) included in the analyses are presented in Table 2. According to their natural biologic behavior, renal neoplasms were divided into indolent (*n* = 14) and aggressive (*n* = 10) groups. There was no evidence of a difference in tumor sizes between the indolent (4.4 cm ± 2.1) and aggressive (6.1 cm ± 3.0) groups (*P* = .12) (Table 2).

### Quantitative MRI for Indolent Renal Neoplasms

**Figure 2:**
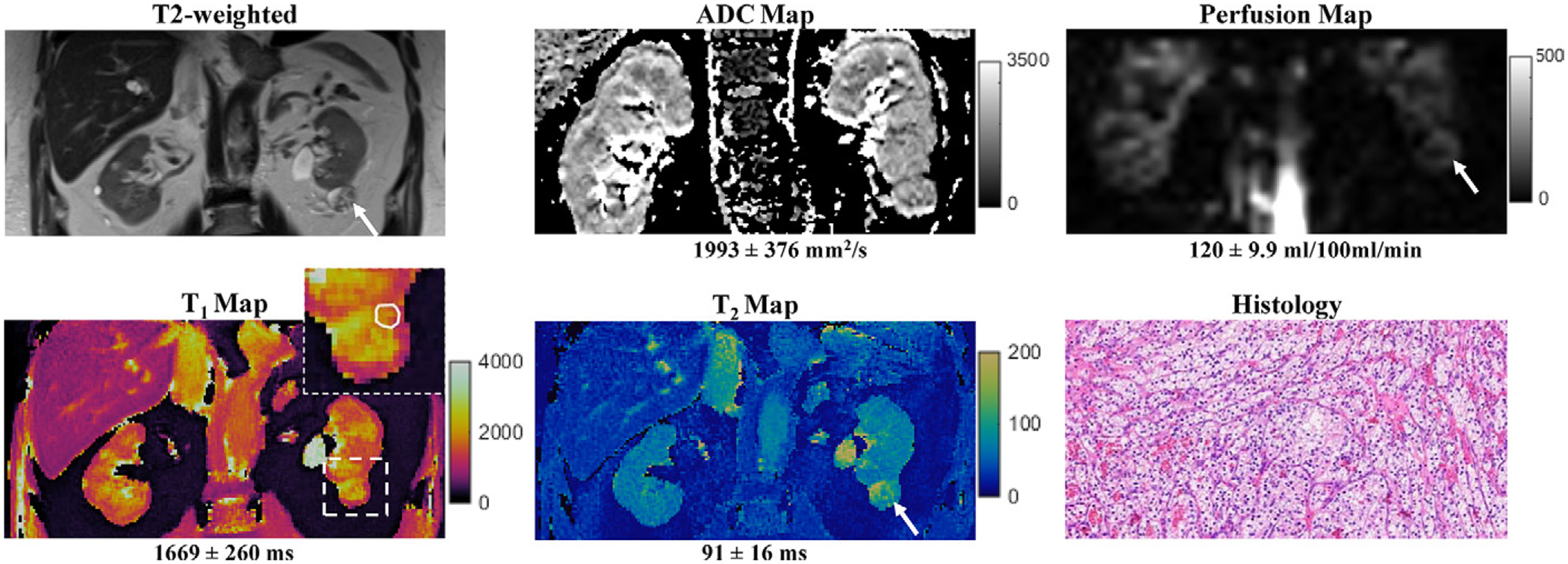
Representative T2-weighted image and quantitative maps (apparent diffusion coefficient [ADC], renal blood flow [ie, perfusion], T1, and T2) in a 75-year-old male participant with grade 2 renal cell carcinoma (arrows; indolent neoplasm). A histologic image with hematoxylin and eosin stain at 20× magnification is also presented. The tumor region of interest is delineated by the solid white outline in the inset indicated by the dashed line. The mean and SD of the quantitative values extracted from the region of interest analysis are shown at the bottom of the maps.

Quantitative MRI including kidney MRF was performed for the 14 participants with indolent renal neoplasms, including 11 low-grade RCCs and three benign oncocytomas. Representative images and maps acquired from one participant with a 2.4-cm grade 2 RCC (indolent neoplasm) are presented in Figure 2. The mean tumor T1 value was 1669 msec ± 260, which is between the T1 values for the normal-appearing renal medulla (2180 msec ± 197) and cortex (1372 msec ± 146) from the contralateral kidney. The tumor T2 value was 91 msec ± 16, whereas the T2 values for the renal medulla and cortex were 70 msec ± 12 and 65 msec ± 10, respectively. The mean RBF was 120 mL/100 mL/min ± 9.9 for the tumor, with a mean ADC of 1993 mm^2^/sec ± 376. Analysis of the histopathologic slide revealed relatively uniform cellular morphologic characteristics and inconspicuous nucleoli.

Representative images in a participant with a chromophobe RCC (5.6 cm) in the right kidney are presented in Figure S2. A more uniform appearance in both T1 and T2 maps was observed for this tumor, with a mean T1 value between that of the normal-appearing medulla and cortex (tumor, 1486 msec ± 137; medulla, 1856 msec ± 122; cortex, 1304 msec ± 125) and a slightly lower T2 value (tumor, 66 msec ± 15; medulla, 76 msec ± 10; cortex, 70 msec ± 10).

The mean T1 and T2 values for the three benign oncocytomas were 1673 msec ± 125 and 86 msec ± 8, respectively, comparable to the values observed for the 11 low-grade RCC tumors (T1, 1602 msec ± 260 [*P* = .66]; T2, 86 msec ± 22 [*P* = .97]).

### Quantitative MRI for Aggressive Renal Neoplasms

**Figure 3:**
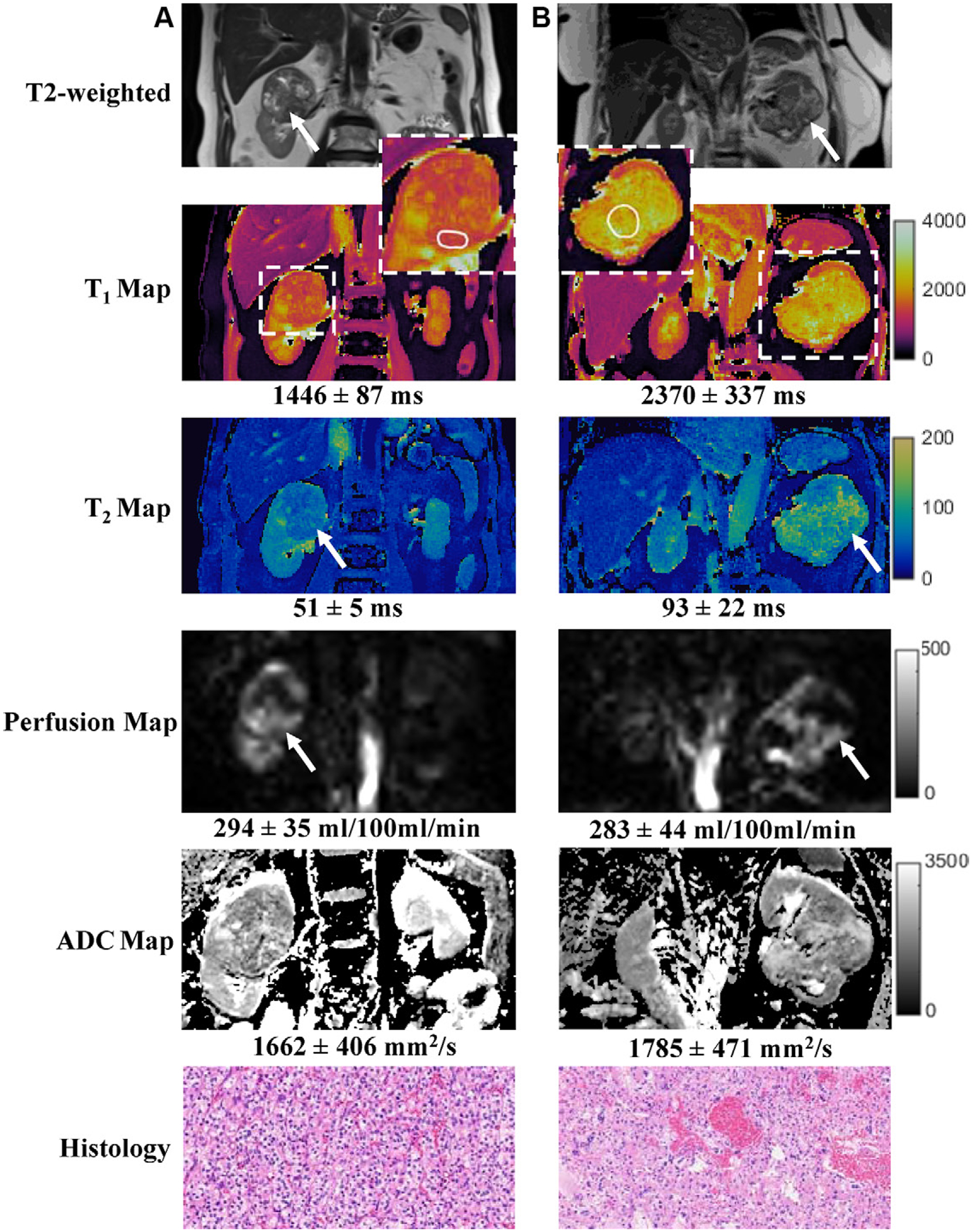
Representative T2-weighted image and quantitative maps (apparent diffusion coefficient [ADC], renal blood flow [RBF] [ie, perfusion], T1, and T2) for two high-grade renal cell carcinomas (RCCs) (arrows; aggressive neoplasms). Images and maps in **(A)** a 68-year-old female participant with a grade 3 RCC and **(B)** an 83-year-old female participant with a grade 4 RCC. Histologic images with hematoxylin and eosin stain at 20× magnification are also presented. The region of interest for each tumor is delineated by the white outline in the inset indicated by the dashed line. The mean and SD of the quantitative values extracted from the region of interest analysis are shown at the bottom of the maps. Although similar mean RBF and ADC values were obtained for the two RCCs, distinct T1 and T2 values were observed.

Representative quantitative tissue property maps for two high-grade RCCs are presented in Figure 3, including maps for a grade 3 RCC (5.8 cm) (Fig 3A) and a grade 4 RCC (8.2 cm) (Fig 3B). Although similar ADC and RBF values were observed, distinct T1 and T2 values were observed for the two tumors. T1 (1446 msec ± 87) and T2 (51 msec ± 5) values were much lower for the grade 3 RCC than the grade 4 RCC (T1, 2370 msec ± 337; T2, 93 msec ± 22). Both RCCs had larger nuclei with more prominent nucleoli than the grade 2 RCC shown in Figure 2. Large areas of necrosis were also noticed in the grade 4 RCC.

Two participants with unclassified RCCs were included in this study, and both RCCs had similar characteristics in terms of quantitative tissue properties (Fig S3). The two unclassified RCCs in general had the lowest T1 and T2 values in the entire sample (T1, 1120 msec ± 177 and 1271 msec ± 190; T2, 38 msec ± 11 and 38 msec ± 18). The histologic slide for one unclassified RCC is included in Figure S3.

### Univariable and Multivariable Analysis

**Figure 4:**
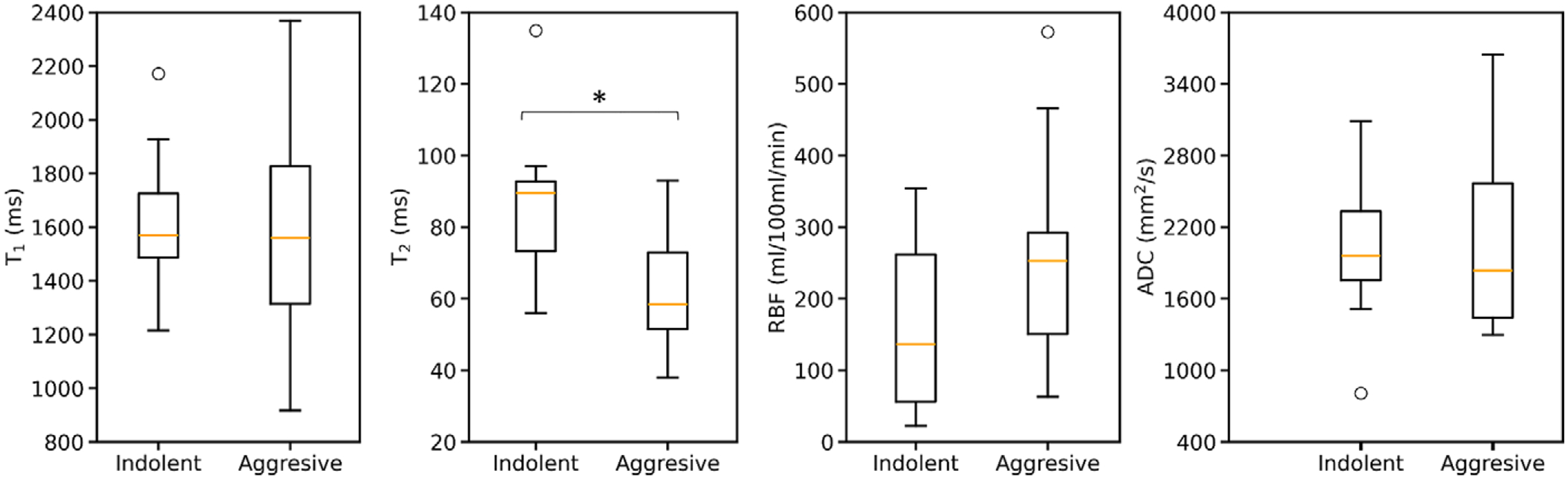
Box plots of T1, T2, renal blood flow (RBF), and apparent diffusion coefficient (ADC) for renal neoplasms. In univariable analysis, a difference in T2 was observed between the indolent and aggressive groups (*P* < .05), whereas no evident difference was noticed in T1, RBF, or ADC values (*P* > .05). The asterisk indicates statistically significant difference. Empty circles represent data points outside 1.5 times the IQR. The orange horizontal line indicates the median, the box borders represent the 25th and 75th percentiles, and the whiskers extend to 1.5 times the IQR.

A summary of the means and SDs of all four tissue properties (T1, T2, ADC, and RBF) is presented in Figure 4. Elevated T2 relaxation time was observed in the indolent neoplasms (86 msec ± 22) compared with the aggressive group (61 msec ± 14; *P* = .005), but there was no evidence of a difference in the other three tissue properties (*P* > .05). Results extracted from prediction models based on both univariable and multivariable analyses are presented in Table 3. In the univariable analysis, MRF-derived T2 provided the best separation between the indolent and aggressive groups, with an AUC of 0.83 (95% CI: 0.50, 1.00). The RBF measurement offered the second-best performance, with an AUC of 0.62 (95% CI: 0.08, 1.00), whereas MRF-derived T1 and ADC assessment showed minimum between-group separation (AUC for T1, 0.39 [95% CI: 0.00, 0.72]; AUC for ADC, 0.38 [95% CI: 0.00, 0.73]).

**Figure 5:**
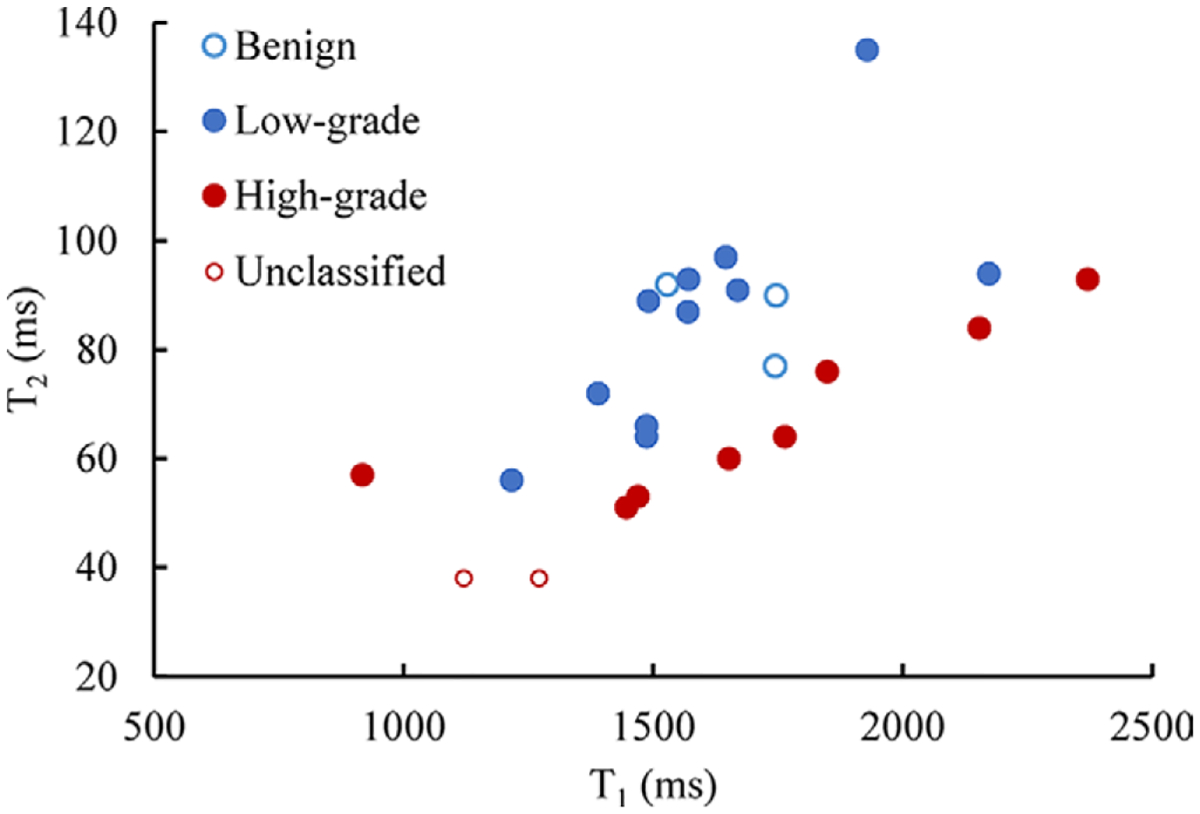
Scatterplot of T1 and T2 relaxation times for all renal masses. Although both the indolent (low-grade and benign) and aggressive (high-grade and unclassified) groups were distributed across a wide range of T1 and T2 values, the two relaxation times derived from kidney MR fingerprinting provide complementary information in better separation of the two tumor groups, with an area under the receiver operating characteristic curve of 0.89.

An exploratory multivariable analysis was performed using the measured tissue properties to differentiate the two renal mass groups. The combination of ADC and RBF showed minimal separation of the two groups, yielding an AUC of 0.51 (95% CI: 0.00, 0.90) (Table 3). In contrast, combining MRF-derived T1 and T2 values provided complementary information and had an AUC of 0.89 (95% CI: 0.60, 1.00; *P* = .06) (Table 3). However, integration of MRF-derived parameters with ADC and RBF did not result in a statistically significant improvement in model performance (*P* > .05). A scatterplot of MRF-derived T1 and T2 values for all the renal masses is presented in Figure 5. When T1 and T2 measurements were combined, the sensitivity and specificity for the differentiation of indolent from aggressive renal neoplasms were 86% and 93%, respectively, demonstrating the strength of multiparametric imaging in effectively differentiating RCCs.

## Discussion

In our initial study, we evaluated the utility of four quantitative MR fingerprinting and MRI assessments, including T1, T2, apparent diffusion coefficient, and renal blood flow, in differentiating renal neoplasms. Among the four tissue properties, T2 relaxation time was the strongest parameter (area under the receiver operating characteristic curve [AUC], 0.83) for distinguishing indolent versus aggressive renal neoplasms in the univariable analysis. In the multivariable analysis, combining T1 and T2 relaxation times had the highest AUC (0.89), providing complementary information in characterizing renal neoplasms.

In earlier studies based on univariable analysis, quantitative T1 and T2 relaxation times were used separately to distinguish RCC tumor grade and biologic aggressiveness (25,26). In comparison with low-grade tumors, high-grade RCC tumors exhibited higher T1 values and lower T2 values, likely owing to upregulation of collagen deposition and higher cellularity (25). In our study, although good agreement in T2 changes based on tumor grade was observed, T1 relaxation time alone provided minimal separation for distinguishing tumor grades. However, the combination of T1 and T2 provided complementary information in tumor characterization, yielding the best separation between indolent and aggressive renal neoplasms. Interestingly, within each tumor group, an increase in T1 was generally associated with an increase in T2. Although the underlying mechanism for this phenomenon remains unclear, it is likely associated with the interplay of multiple underlying biologic and physiologic factors at the tissue level.

It is well established that both T1 and T2 relaxation times are affected by various tissue properties, including cellular density, water content, macromolecule concentration, and iron deposition. According to the International Society of Urological Pathology grade definition, high-grade RCCs often exhibit enlarged nuclei and pronounced nucleoli, generally leading to notably lower T1 and T2 values (27). Additionally, the increased collagen content in the extracellular environment of high-grade RCCs also contributes to lower relaxation times (28). However, depending on tumor stages, high-grade and aggressive RCCs are often associated with necrosis, angiogenesis, hemorrhage, and increased extracellular matrix (29,30). These pathophysiologic factors all result in increased water content within the lesion, which can lead to an increase in T1 and T2.

Conventional quantitative MRI techniques typically measure kidney T1 and T2 alone or sequentially, with different spatial resolutions and separate breath holds for each measurement. Achieving precise alignment of these separately acquired T1 and T2 maps to capture simultaneous multiparametric variation is technically challenging. In contrast, the MRF-derived T1 and T2 maps are both acquired in a single breath hold and are inherently coregistered, providing a unique opportunity to capture the interplay of multiple tissue components in renal masses and thus largely improve MRF performance in tumor characterization. Future experiments will be performed to coregister MRF maps with histopathologic slides of renal masses to examine this hypothesis at the microscopic tissue level. In addition, the accuracy and robustness of the kidney MRF technique have been systematically evaluated in both phantom and in vivo experiments. Compared with conventional methods, another advantage of kidney MRF is its high accuracy and reproducibility (<3% variability for both T1 and T2) and its inherent resistance to errors from B_1_ field inhomogeneities and respiratory motion artifacts, enabling precise and comprehensive characterization of renal tissues (13). Nonetheless, the potential advantages of MRF observed in this small exploratory study require confirmation in adequately powered validation studies before definitive conclusions can be drawn about its clinical utility.

Previous studies have also investigated the utility of diffusion MRI and ASL MRI in characterizing renal masses. A study of over 100 renal lesions revealed significantly lower ADCs in malignant lesions than in benign lesions (31). In addition, significantly higher ADCs were observed for high-grade versus low-grade RCCs, suggesting its potential in tumor grade differentiation for RCCs (6–8). ASL MRI has also been applied in distinguishing different renal mass subtypes according to perfusion level (9). However, the acquisition time is relatively long for diffusion MRI and ASL MRI, and both techniques are highly susceptible to respiratory motion artifacts. Although respiratory gating and motion correction are typically applied, the acquired quantitative measures often exhibit poor reproducibility (32), limiting their utility in renal tissue and tumor characterization and differentiation. In this exploratory study, integrating MRF measures with ADC and RBF did not demonstrate statistically significant value in renal mass differentiation, which may be attributable to these technical limitations, the small sample size, or both. Larger studies with optimized acquisition protocols are needed to definitively assess the relative contributions of diffusion and perfusion imaging.

Our initial study has several limitations. First, the sample size is small (*n* = 24), which was constrained by the feasibility of recruiting patients with suspected renal cancer to undergo both experimental MRF imaging and surgical resection within the study timeframe. This limited statistical power to detect differences between imaging parameters and models, as evidenced by wide confidence intervals (eg, 95% CI: 0.60, 1.00 for the AUC of the T1 + T2 model) and lack of statistically significant difference in the multivariable model comparisons (*P* > .05). Although bootstrap validation with optimism correction was used to provide realistic performance estimates, this cannot overcome fundamental sample size limitations. Accordingly, all results are exploratory and hypothesis generating rather than definitive, and validation in larger, independent cohorts is essential before clinical translation can be considered. Most of the enrolled participants underwent surgical resection, which might have introduced a selection bias toward more aggressive tumors. However, the fact that over 50% of the enrolled participants had either benign or low-grade renal masses underscores the clinical significance of developing novel, noninvasive methods for differentiating tumor aggressiveness in kidney cancer. Second, the region of interest analysis was performed by one experienced radiologist, and the quantitative metrics were extracted from the solid tumor on the basis of the perfusion measurement. Earlier studies have shown variations when selecting solid tumor versus whole tumor (30). How to precisely select regions of interest is still an open question. When sufficient patient data are available, the development of a deep learning autosegmentation framework has great potential to eliminate this time-consuming process, resulting in improved consistency and robustness in image postprocessing and analysis. Third, we focused on mean tissue properties extracted by means of region of interest–based analysis. More advanced approaches, such as radiomics models, could provide additional information to improve tissue and tumor characterization. Fourth, robust registration of ADC and RBF maps to MRF maps is technically challenging owing to different tissue contrasts, image resolution, coverage, and quality. Therefore, the regions of interest for ADC and RBF were drawn separately, which might have introduced additional variation in the multiparametric analysis. Imaging registration could be explored when the map quality for ADC and RBF is substantially improved, especially when coupled with robust motion management strategies.

In conclusion, we evaluated the application of kidney MR fingerprinting (MRF) in characterizing the aggressiveness of renal neoplasms, and our preliminary results indicate that MRF-derived T1 and T2 relaxation times may provide complementary information in the differentiation of indolent versus aggressive renal masses. The developed approach has great potential for eliminating unnecessary biopsies or surgeries in eligible patients with low-grade or benign renal masses and for providing guidance to determine the most appropriate treatment strategy.

## Supplementary Material

Supplemental Material

## Figures and Tables

**Table 1:** Acquisition Parameters for the Imaging Protocol, Including T1-weighted, T2-weighted, Kidney MRF, DWI, and ASL MRI

Parameter	T1-weighted	T2-weighted	MRF	DWI*	ASL
Acquisition sequence	Gradient echo	HASTE	FISP	Multishot EPI	Gradient echo
Field of view (mm)	400 × 400	400 × 400	400 × 400	240 × 240	320 × 160
Matrix size	300 × 300	300 × 300	256 × 256	160 × 160	64 × 32
Spatial resolution (mm)	1.3 × 1.3	1.3 × 1.3	1.6 × 1.6	1.5 × 1.5	5.0 × 5.0
Section thickness (mm)	3	5	5	4	5
Repetition time (msec)	9.0	1050	5.7	2400	5000
Echo time (msec)	1.1	82	1	55/91	19.2
Flip angle (degrees)	4	…	5–12	…	…
Duration (min)	0.25	0.32	0.25 per section	…	5.3

Note.—ASL = arterial spin labeling, DWI = diffusion-weighted imaging, EPI = echo-planar imaging, FISP = fast imaging with steady-state free precession, HASTE = half-Fourier acquisition single-shot turbo spin echo, MRF = MR fingerprinting.

* Two *b* values (0 and 400 sec/mm^2^) were applied with 12 diffusion directions.

**Table 2:** Participant Characteristics

Characteristic	Total (*n* = 24)	Indolent Neoplasm Group (*n* = 14)	Aggressive Neoplasm Group (*n* = 10)	*P* Value*
Age (y)^†^	61 ± 11	58 ± 10	64 ± 13	.21
Sex				
M	8	4	4	…
F	16	10	6	…
Interval between MRI and surgery (d)^‡^	10 (5–15)	11 (7–14)	9 (4–22)	.52
Laterality				
Left	16	8	8	…
Right	8	6	2	…
Tumor size (cm)^†^	5.1 ± 2.6	4.4 ± 2.1	6.1 ± 3.0	.12

Note.—Unless otherwise specified, data are numbers of participants.

* No statistically significant difference was observed between the indolent and aggressive groups (*P* > .05).

† Data are means ± SDs.

‡ Data are medians, with IQRs in parentheses.

**Table 3:** Parameters Evaluated in Univariable and Multivariable Analysis and Their Performance Metrics in the Differentiation of Indolent Versus Aggressive Renal Neoplasms

Analysis and Model	AUC	*P* Value*	Sensitivity (%)	Specificity (%)
Univariable analysis				
ADC	0.38 (0.00, 0.73)	…	39	64
RBF	0.62 (0.08, 1.00)	.20	75	59
T1	0.39 (0.00, 0.72)	.54	39	68
T2	0.83 (0.50, 1.00)	.02	82	83
Multivariable analysis				
ADC + RBF	0.51 (0.00, 0.90)	…	60	59
T1 + T2	0.89 (0.60, 1.00)	.06	86	93
ADC + RBF + T1	0.42 (0.00, 0.80)	.50	49	57
ADC + RBF + T2	0.75 (0.38, 1.00)	.13	80	73
ADC + RBF + T1 + T2	0.83 (0.50, 1.00)	.07	79	87

Note.—Data in parentheses are 95% CIs. MR fingerprinting–derived T1 and T2 relaxation times provided complementary information, yielding an area under the receiver operating characteristic curve (AUC) of 0.89 in this analysis. ADC = apparent diffusion coefficient, RBF = renal blood flow.

* *P* values for the AUC comparison are presented, using the ADC-only model as the reference for the univariable analysis and the ADC + RBF model as the reference for the multivariable analysis.

## Data Availability

Data generated or analyzed during the study are available from the corresponding author by request.

## Key Results

- In a prospective study of 24 participants with renal cancer, T2 performed best (area under the receiver operating characteristic curve [AUC], 0.83) for differentiating indolent and aggressive renal neoplasms in univariable analysis (AUCs: T1, 0.39; apparent diffusion coefficient, 0.38; renal blood flow, 0.62).
- In multivariable analysis, MR fingerprinting–derived T1 and T2 combined provided complementary information in renal neoplasm characterization, with an AUC of 0.89 in tumor type differentiation.

## Abbreviations

ADC: apparent diffusion coefficient
ASL: arterial spin labeling
AUC: area under the receiver operating characteristic
MRF: MR fingerprinting
RBF: renal blood flow
RCC: renal cell carcinoma
