# Supplementary material for: Noninvasive Differentiation of Indolent from Aggressive Renal Neoplasms with MR Fingerprinting Combined with Diffusion/Perfusion MRI
Source: Radiology. Author manuscript; Available in PMC 2026 Jul 6. (PMC13335708; doi:10.1148/radiol.251404)
Supplement: Supplemental Material [file NIHMS2192274-supplement-Supplemental_Material.pdf]

Supplemental materials undergo peer review but are not copyedited, and may include typographical errors.

©The Author(s) 2026. Published by the Radiological Society of North America under a CC BY 4.0 license.
10.1148/radiol.251404

Appendix S1

METHODS

Image Reconstruction and Processing

Magnetic Resonance fingerprinting (MRF) reconstruction and image post-processing were performed offline on a standalone workstation (12 core, 2.6-GHz Intel Xeon E5-2630 v2 processor; 128 GB RAM). Non-uniform Fourier transform was first performed to reconstruct the series of MRF time frames. The obtained signal evolution from each pixel was then matched to the MRF dictionary to extract the corresponding T_1 and T_2 relaxation times (**Figure S1**). The dictionary entries were pre-calculated by using Bloch equation simulations with a T_1 range of 0-5000 ms and T_2 range of 0-700 ms, yielding ~54,000 dictionary entries. In addition to T_1 and T_2 relaxation time maps, the apparent diffusion coefficient (ADC) maps from diffusion MRI and renal blood flow (RBF) maps were also generated inline after data acquisition. 3D motion correction was performed for the pseudo-continuous arterial spin labeling (ASL) scans to reduce artifacts introduced by respiratory motions.

Following the same guidelines introduced for the region-of-interest (ROI) analysis, an MR physicist with 12 years of experience in kidney imaging, who was blinded to the prior ROI analysis results, independently redrew the ROIs for all four tissue property maps. The extracted values were used to evaluate interreader variability in quantitative assessment but not to characterize tumors in this study. Both Pearson correlation coefficients and intraclass correlation coefficients were calculated for the statistical analysis.

RESULTS

Reader Agreement Assessment

Interreader variability was assessed for all four quantitative tissue properties across the 24 renal neoplasms. MRF-derived T_1, T_2, and RBF measurements yielded Pearson correlation coefficients (r) of 0.80, 0.81, and 0.91, respectively, and intraclass correlation coefficients of 0.82, 0.83, and 0.91, respectively. In comparison, slightly lower agreement was noted for ADC values (Pearson correlation coefficient [r] = 0.69; Intraclass correlation coefficient = 0.72).

Supplemental materials undergo peer review but are not copyedited, and may include typographical errors.

DISCUSSION

In addition to quantitative MRI, the diagnosis and characterization of renal masses via other techniques have been explored. Findings from a phase 3 clinical trial revealed that PET-CT imaging with [^{89}Zr]Zr-girentuximab can achieve 85.5% sensitivity and 87% specificity in the differentiation of clear-cell from non-clear cell RCC lesions (1). Radiomics models built on conventional MRI scans, including fat-suppressed T_2-weighted, contrast-enhanced T_1-weighted, and DWI, have been shown to provide a sensitivity of 77%, a specificity of 80%, and an area under the receiver operating characteristic curve (AUC) value of 0.84 in differentiation of high-grade versus low-grade clear-cell RCCs (2). While MRI is generally preferred for patients with kidney cancer because of its role in staging and surgical planning, its findings, including T_1 and T_2 measurement from kidney MRF, can be integrated with other imaging modalities and advanced imaging processing techniques to further improve its overall performance in characterization of tumor subtypes and grades for renal masses. In routine clinical practice, tumor size is sometimes used as an indicator for surgery, irrespective of tumor type. Although no statistical difference in tumor size was observed between the indolent and aggressive renal neoplasms in our study, such clinical information will be incorporated in future work to improve renal mass characterization.

REFERENCES

1. Shuch B, Pantuck AJ, Bernhard J, et al. [^{89}Zr] Zr-girentuximab for PET – CT imaging of clear-cell renal cell carcinoma : a prospective , open-label , multicentre , phase 3 trial. Lancet Oncol. 2024;25(10):1277–1287.

2. Li Q, Liu Y jia, Dong D, et al. Multiparametric MRI Radiomic Model for Preoperative Predicting WHO/ISUP Nuclear Grade of Clear Cell Renal Cell Carcinoma. J Magn Reson Imaging. 2020;52(5):1557–1566.

Supplemental materials undergo peer review but are not copyedited, and may include typographical errors.

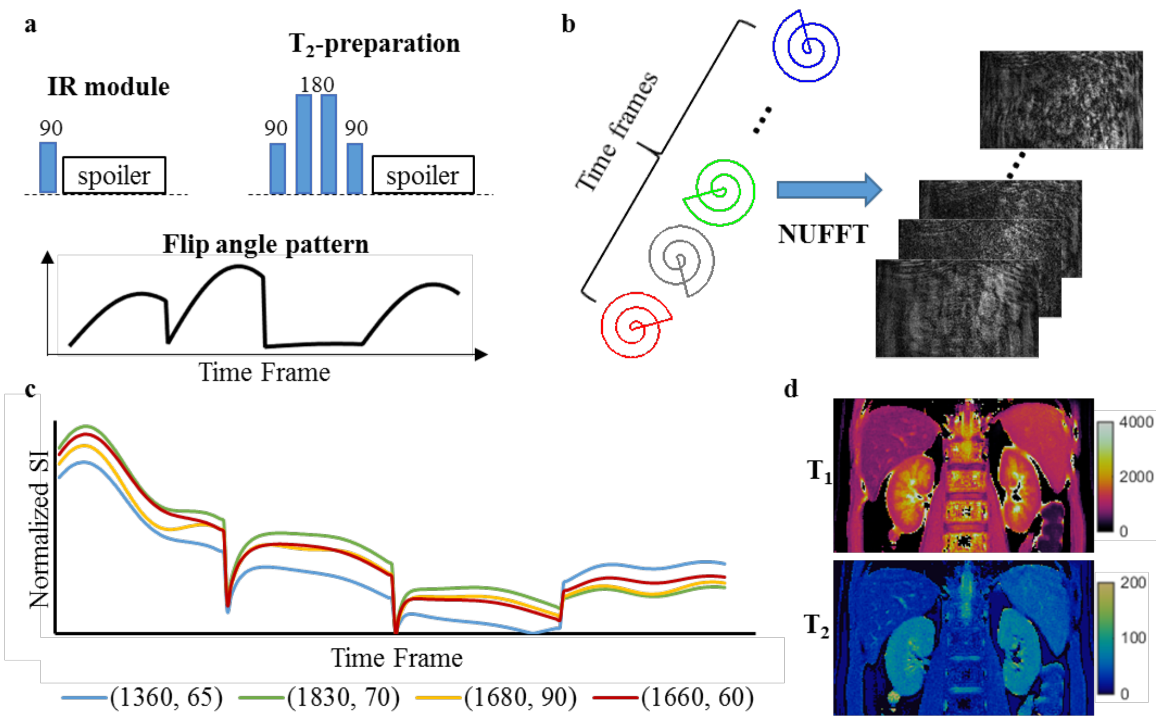

Figure S1: Schematic drawing of the kidney Magnetic Resonance Fingerprinting (MRF) technique. (a) Multiple preparation modules including inversion recovery (IR) and T_2-preparation modules are included in the MRF acquisition to improve its performance in assessing T_1 and T_2 values in renal tissues/tumors. A total of 1728 time frames were acquired in one MRF scan with variable flip angles. (b) Each time frame was acquired with one spiral interleaf and non-uniform fast Fourier transform was applied to reconstruct the MRF time frames. (c) Representative MRF signal evolutions for four pairs of (T_1, T_2) values. (d) Representative T_1 and T_2 maps obtained from a healthy subject.

Supplemental materials undergo peer review but are not copyedited, and may include typographical errors.

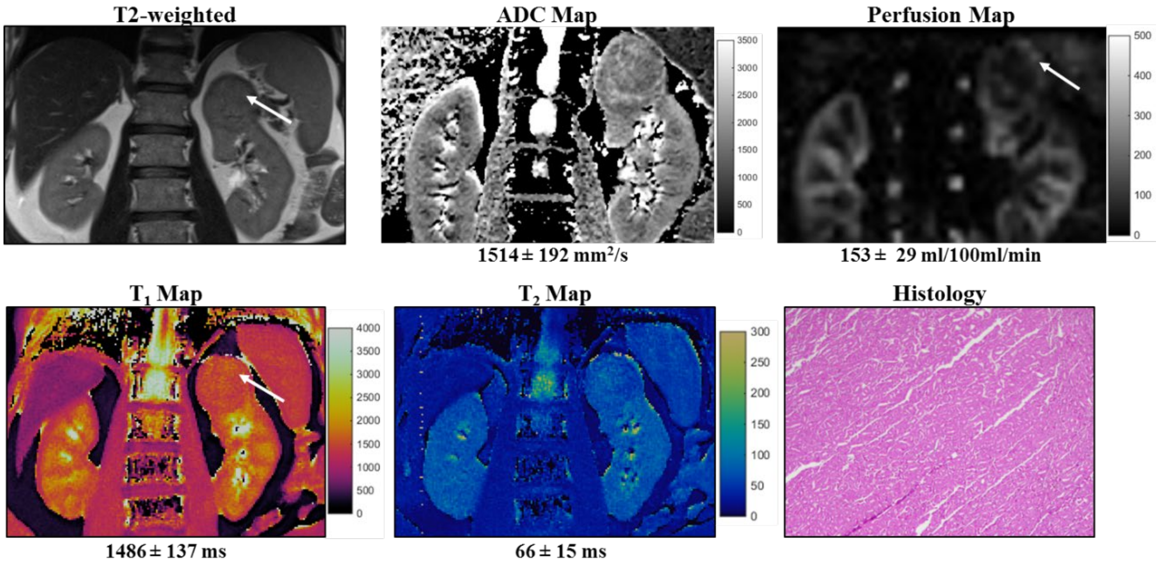

Figure S2: Representative T_2-weighted image and quantitative maps (apparent diffusion coefficient (ADC), renal blood flow (RBF), T_1, and T_2) obtained from a 55-year-old male with chromophobe renal cell carcinoma (RCC; indolent neoplasm). Mean and standard deviation of the quantitative values extracted from the region-of-interest (ROI) analysis are listed at the bottom of the maps. Histological image with hematoxylin and eosin stain at 20× magnification is also presented.

Supplemental materials undergo peer review but are not copyedited, and may include typographical errors.

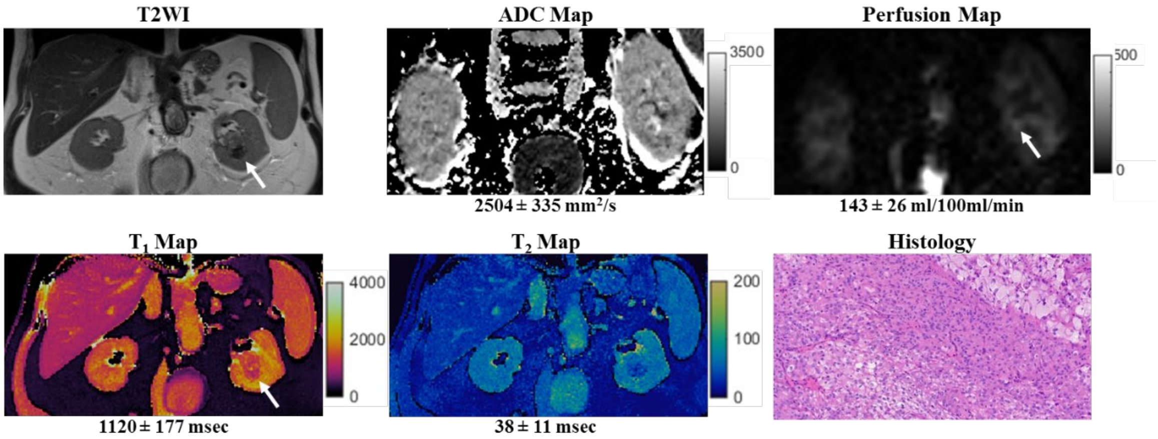

Figure S3: Representative T_2-weighted image and quantitative maps (ADC, RBF, T_1, and T_2) obtained from a 68-year-old male with unclassified RCC (aggressive neoplasm). Mean and standard deviation of the quantitative values extracted from the ROI analysis are listed at the bottom of the maps. Histological image with hematoxylin and eosin stain at 20× magnification is also presented.
